# Formulaire Des Medications Nouvelles

**Published:** 1896-07

**Authors:** 


FORMULAIRE DES MEDICATIONS NOUVELLES, par le DR. H. GILLET, ancien interne des hôpitaux de Paris, chef du service des maladies des enfants à la Polyclinique de Paris. 1 vol. in 18 de 280 pages, avec fig., cart. 3 fr. Paris: J.-B. Baillière et Fils, 19 rue Hautefeuille (près du boulevard Saint-Germain). 1896.

This little book, published only in the original French, contains much valuable information regarding applied medicines, particularly local antiseptics and injections of serum of various kinds, antitoxines and medicinal agents, such as the soluble mercurial salts, applied subcutaneously, by intravenous injections, and directly to the organs themselves as intrapulmonary injections. There are several interesting sections on the different extracts, and juices, such as thyroid extract, testicular extract or Sequardine. The articles are condensed and very systematically arranged. Under the title Principe de la methode is explained the direct idea which has given birth to the new medication; then are indicated the nature of the medication, the mode of administration and doses ; in some instances the effects and contraindications are given, and some formulæ.

Among the most instructive articles are those under the following headings : Antisepsis of the upper digestive and respiratory tracts ; Antisepsis of the nasal fossæ ; Antisepsis of the stomach and intestines ; Lavage of the stomach ; Antisepsis of the skin ; Cold baths ; Intrapulmonary injections ; Anti-tuberculous serums ; Sequardine or testicular extract. J. N. F.

